# Investigating Global Lipidome Alterations with the Lipid Network Explorer

**DOI:** 10.3390/metabo11080488

**Published:** 2021-07-28

**Authors:** Nikolai Köhler, Tim Daniel Rose, Lisa Falk, Josch Konstantin Pauling

**Affiliations:** LipiTUM, Chair of Experimental Bioinformatics, TUM School of Life Sciences, Technical University of Munich, 85354 Freising, Germany; nikolai.koehler@tum.de (N.K.); tim.rose@wzw.tum.de (T.D.R.); lisa.falk@tum.de (L.F.)

**Keywords:** computational lipidomics, computational systems biology, network biology, bioinformatics, lipidomics, lipids, metabolic networks

## Abstract

Lipids play an important role in biological systems and have the potential to serve as biomarkers in medical applications. Advances in lipidomics allow identification of hundreds of lipid species from biological samples. However, a systems biological analysis of the lipidome, by incorporating pathway information remains challenging, leaving lipidomics behind compared to other omics disciplines. An especially uncharted territory is the integration of statistical and network-based approaches for studying global lipidome changes. Here we developed the Lipid Network Explorer (LINEX), a web-tool addressing this gap by providing a way to visualize and analyze functional lipid metabolic networks. It utilizes metabolic rules to match biochemically connected lipids on a species level and combine it with a statistical correlation and testing analysis. Researchers can customize the biochemical rules considered, to their tissue or organism specific analysis and easily share them. We demonstrate the benefits of combining network-based analyses with statistics using publicly available lipidomics data sets. LINEX facilitates a biochemical knowledge-based data analysis for lipidomics. It is availableas a web-application and as a publicly available docker container.

## 1. Introduction

Lipids play a central role in biology for membranes, energy metabolism and signaling processes. Lipidomics is gaining impact in systems biology and medicine as lipids are an important molecular dimension for the investigation of biological mechanisms, stratification of patients, and disease subtyping. Recent advances in extraction protocols, high resolution Mass Spectrometry (MS) and methods for the identification and quantification of lipids allow for more comprehensive and complex lipidomes to be measured. However, the analysis of lipidomics data does not end with quantification. To interpret changes of the lipidome and embed them into a systems biological context, dedicated computational approaches are necessary. The software tools lipidr [1] and LipidSuite [2] provide statistical methods to mine and perform differential analysis of lipidomics data. They implement a “Lipid Set Enrichment Analysis” and “Lipid chain analysis” to investigate the regulation of lipid classes, carbon chains or saturations. These approaches incorporate lipid-specific characteristics into the statistical analysis. However, the possibility to investigate associations between lipids is missing.

Association networks from molecular omics data can offer benefits for data analysis, as biological networks carry information about functional interactions of biomolecules. Examples are Protein-Protein Interaction (PPI) networks, Gene Regulatory (GR) networks, or metabolic networks. In the case of lipid metabolic networks, these characterize transformations of lipids catalyzed by enzymes. Dedicated bioinformatics tools such as KeyPathwayMiner [3,4], DOMINO [5] or HotNet2 [6] have been developed, which extract functionally associated network modules enriched with deregulated genes/proteins from PPI networks in a case/control setting. Such network modules can hint towards biochemical mechanisms, which connect a phenotype to its underlying molecular machinery. Applying network-based computational methods on lipidomics data remains challenging. One reason is that reaction databases carry information mainly on a lipid class level but not on a molecular species level [7,8]. Since modern lipidomics experiments provide measurements on the sum or molecular species level, more fine-grained reaction information can be utilized. Therefore, (partial) correlation networks of lipids species can be used to investigate data-driven interactions between lipids.

Correlation networks are a common method for the analysis of metabolomics/lipidomics data [9,10,11]. They show relationships between lipids entirely based on pairwise correlations over all measured samples. While they can reveal novel relationships between lipids, they do not describe functional associations between them. Recently it was shown that correlation networks can profit from incorporating prior knowledge into cut-off selection [12], providing an alternative to purely data-driven or purely knowledge-driven metabolic networks. An interplay between functional and data-driven associations could therefore be beneficial for the analysis of lipidomics experiments.

Functional analysis of lipid data is already possible with tools such as LION/web [13] or BioPAN [14], which enrich lipids based on an ontology or pathways. LION/web identifies lipid-associated terms in lipidomes [13] and associates biological functions to lipidomics data. BioPAN visualizes biochemical pathways of lipids, which can be investigated on the lipid class, species or fatty acid (FA) metabolism level. Additionally, BioPAN provides quantitative scores for the activity of pathways. However, they focus on the enrichment of pathways or reaction chains rather than on a global analysis of the lipidome.

Another approach for the global qualitative analysis of the lipidome is the LUX Score [15]. The methodology embeds the lipidome in a chemical space, such that lipids are close to each other if they exhibit a high chemical similarity (based on SMILES notation of chemical structures). The LUX Score also operates on the lipid species level. It provides an overview of chemical properties and a qualitative comparison of lipidomes.

Here we present the Lipid Network Explorer (LINEX), a flexible web-application (app) to create, visualize and analyze functional lipidomics networks. It combines enzymatic transformations between lipids with correlations and statistical properties that can be superimposed onto the network. This enables a global and a local view on the lipidome. The tool thereby provides a basis for introducing graph-theoretical and network-topological approaches into the analysis of lipidomics data. We further present applications of LINEX on available lipidomics data sets and show the benefits of a network-based analysis.

## 2. Results

**Figure 1:**
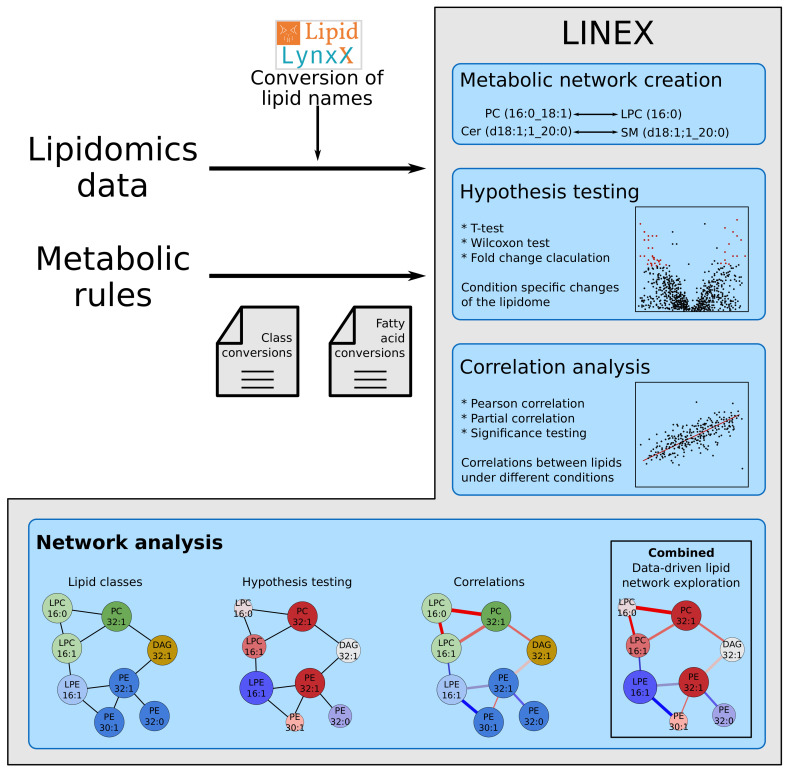
Workflow of the LINEX approach. Lipidomics data and optionally customized metabolic rules are uploaded by the user. The data are used to generate an experiment-specific lipid network, which can be visualized together with statistical measures such as correlation and fold change.

We developed LINEX to visualize and analyze functional associations of lipids on networks (Figure 1), enabling the investigation of lipidomics data in the context of metabolic reactions. In such networks, lipids are represented as nodes, while edges indicate a connection via enzymatic reactions of lipid classes or FAs (Figure A2a in Appendix A). These reactions are encoded as rules customizable by the user. This way, condition-, tissue-, or organism-specific lipid metabolic properties can be incorporated into an analysis with LINEX. As default settings, common reactions of glycero-, glycerophospho- and sphingolipids as well as typical FA modifications are included. LINEX then combines reactions of lipid class and FA metabolism into one network to give a comprehensive overview of lipid species metabolism.

On the basis of experimental lipidomics data, and optional sample group annotation, data specific metabolic networks are computed. Supported by a data driven lipid network exploration, correlation analysis and hypothesis testing can be added to the network representation (Figure 1) for a combined analysis.

LINEX is available as a web-app (https://exbio.wzw.tum.de/linex/ (accessed on 27 July 2021)), where lipidomics data can be uploaded (Figure A2a), networks computed and interactively visualized (Figure A2b). The lipidomics data have to be uploaded as one table (data from two ion modes have to be processed and combined by the users to one table prior to the analysis with LINEX). Additionally, the networks and all computed statistical measures can be downloaded (Figure A2c). In the following, we apply LINEX to three publicly available lipidomics datasets. They were selected to cover technical aspects such as MS1, MS2 and lipidome coverage and experimental designs such as case-control, time series and multi-group conditions. On those, we present our workflow to analyze combined metabolic and data driven lipid networks.

All networks shown in the results section are available as interactive HTML files (Supplementary Data 1–3).

### 2.1. Lipidomics of Colorectal Cancer

We investigated lipidomics data from Wang et al. [16] about a lipidomics characterization of colorectal cancer patients. The authors identified and quantified 342 lipid species from 20 different lipid classes. According to the authors, no global changes of the lipidome were detected, but alterations in individual lipids were observed.

**Figure 2:**
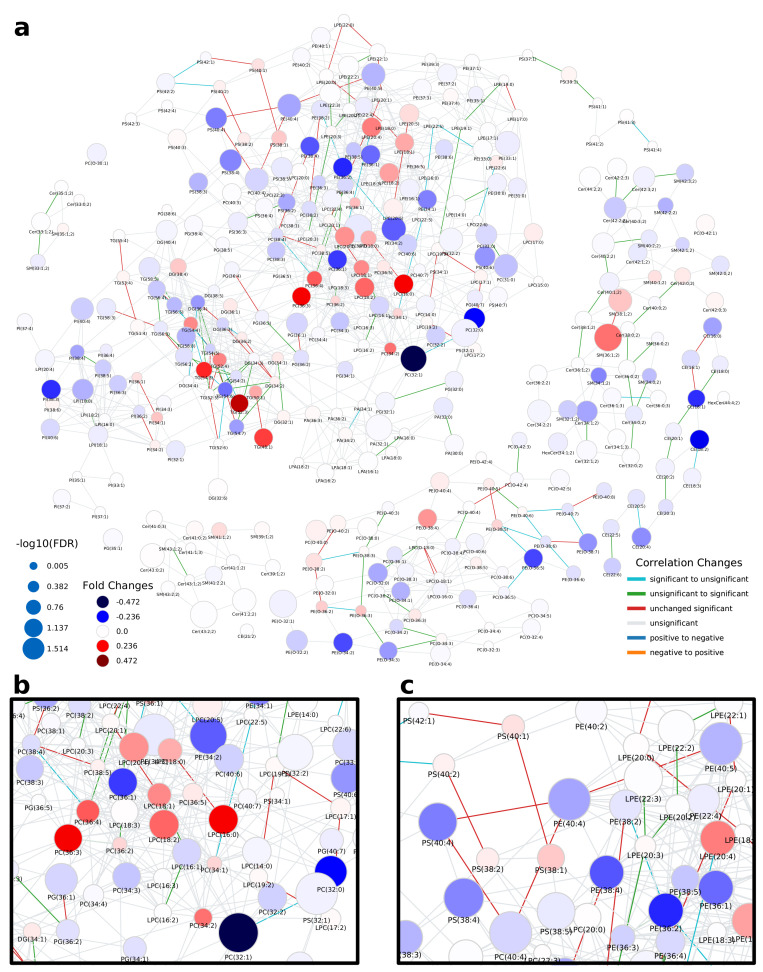
Lipid network of colorectal cancer lipidomics data from Wang et al. [16]. (**a**) Full lipid network with node size scaled by negative log10 of *p*-values for comparison between healthy and cancer tissue. Lipids are colored by log fold change between healthy and cancer tissue. Blue colors indicate lower levels of lipids in the healthy condition compared to the tumor and red higher levels in healthy samples. Edges are colored by changes of correlation for lipids from the healthy to cancer condition. For example, green indicates a non-statistically significant correlation in the healthy condition and a statistically significant correlation in the tumor, where the correlation has the same sign. (**b**) Subnetwork showing PC and LPC nodes. (**c**) Subnetwork showing mainly unsaturated glycerophospholipids.

The network computed by LINEX (Figure 2a, interactive network: Supplementary Data 1) shows a global view on the changes of the lipidome between colorectal tumor and normal mucosa. In the network, each node represents a lipid species, and each edge between a pair of lipids indicates a biochemical reaction capable of transforming the lipids into each other on the class or FA level. Edges are colored by changes of correlation from healthy to cancer condition. Node colors represent the log fold change between healthy and cancer samples, with red indicating increased and blue indicating decreased lipid levels under healthy conditions. Node sizes indicate the negative log10 FDR-values of a lipid between the two conditions, where more strongly altered lipids are displayed as larger nodes.

At first glance, it can be observed that the majority of reactions (edges) between lipid species do not represent significant correlations in either of the two conditions (FDR < 0.05, used throughout the manuscript as the significance cut-off). However, highly intraconnected parts of the network (local communities) can be observed, which exhibit significant correlations, indicated by colored edges. Some examples are triacylglycerol (TG) and diacylglycerol (DG) species (Figure A3a). While the fold changes of individual species are heterogeneous, a trend of higher unsaturated TG species increasing in tumor tissue and higher saturated TG species decreasing is observable. In particular, correlations between highly unsaturated TGs (52:5, 54:5, 54:6, 54:7) remain significant over both conditions, while others occur (green) or disappear (cyan) when comparing normal mucosa to tumor mucosa. This indicates changes in the regulation of the FA metabolism related to neutral lipids.

A big part of the network comprises the metabolism of GPLs. The network shows a set of phosphatidylcholine (PC) and lyso-phosphatidylcholine (LPC) species, which decrease in tumor samples and are metabolically closely related via reactions catalyzed by the MBOAT7 and PLA2 enzymes (Figure 2b). MBOAT7 expression has previously been associated with gastrointestinal cancer risk [17] as well as lipid-linked liver diseases [18], which we were able to link to lipidome alterations by only considering the LINEX network. The respective set of lipids is surrounded by PC, phosphatidylethanolamine (PE) and LPC species, which show the opposite behavior. We could also observe an interesting pattern of correlation of poly-unsaturated GPLs (Figure 2c). Here, PC, phosphatidylserine (PS) and PE species which have a sum composition of 40:4, and were all found to be significantly upregulated in the original publication additionally show functional correlations between each other, independent of the condition. This is a strong indication of a common mechanism regulating these lipid species.

In the metabolism of phosphatidylinositol (PI), high fold changes could be observed in poly-unsaturated PI species, while some highly connected lyso-phosphatidylinositol (LPI) species 18:2 and 16:0 did not seem to be influenced by the tumor (Figure 2a, left). The authors argued that ether lipids might play a role in tumor progression, especially lower levels of phosphatidylethanolamine ether (PEO) indicating higher oxidative stress. Our analysis shows a close biochemical connection between downregulated PEO species (Figure A3c). Other PEO species (e.g., PE(O-38:5) to PE(O-36:5), or PE(O-40:6) to PE(O-40:7)), which increase in the tumor condition only show significant correlation in healthy samples, revealing a diverging pattern in ether-PE. A reaction chain of ceramides with significant correlations could be observed in the sphingolipid metabolism component of the network (Figure A3b). While the Cers themselves are not significant, their correlations show a clear co-regulation. This shows that changes of individual lipids might not always be significant, but a combined network analysis with functional interactions and correlations can nevertheless reveal interesting relations between lipids as well as indicate putative common regulatory mechanisms.

### 2.2. Lipidome Alterations in Aging Brain of Mice

**Figure 3:**
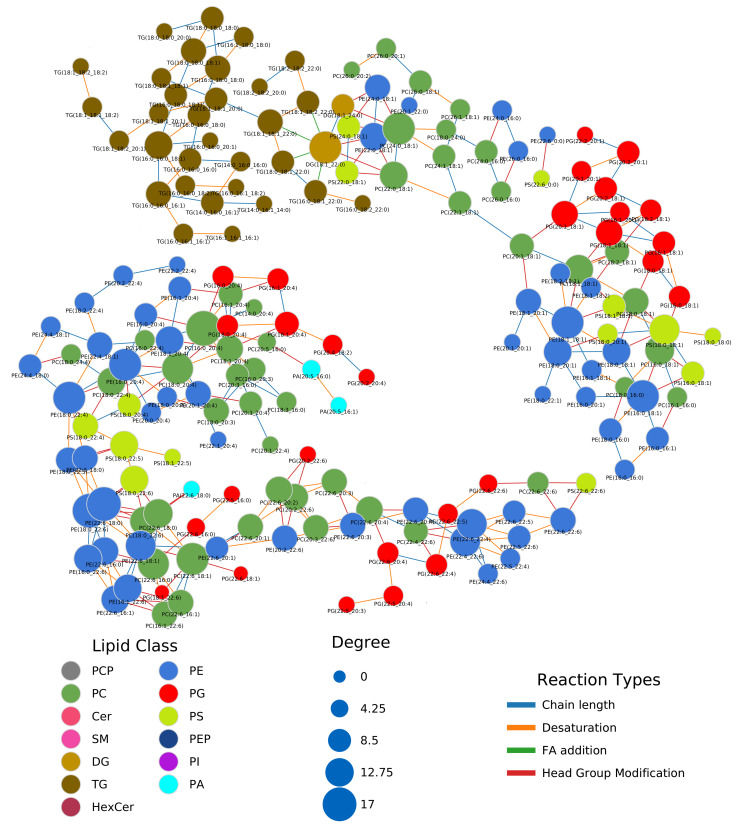
Part of the lipid network of the lipidomics data from Tu et al. [19]. Shown are the two main components of the GPL metabolism. Nodes are colored by lipid class, and edges are colored by reaction type. Node sizes represent the degree.

Next, we investigated a lipidomics experiment from Tu et al. [19] about lipidome changes in the aging brain of mice between the age of 4 weeks to 52 weeks. Although not compatible with the LipidLynxX [20] converter, we manually added Sulfatide and Hex2Cer to the metabolic rules. In contrast to the previous data set, we could observe very few correlations between lipids (Figure A4). To standardize the coloring of lipids in networks, we developed a unified color scheme on the lipid class level (see Section 4). The types of reactions forming edges between lipids are mainly chain length modifications and desaturations. Lipid headgroup modifications can be observed primarily between GPLs (Figure 3, interactive network: Supplementary Data 2). FA additions/removals are only found between DG(18:1_22:0) and three TG species. Figure 3 shows a subnetwork of highly saturated TG species, which are only connected via FA reactions. We first observed a decrease of TG species from 4 to 12 weeks, followed by a strong increase of TG levels starting from the age of 32 weeks. This may be an indication for increased de novo lipogenesis, which might be explained with FAS (fatty acid synthase, preferentially synthesizes palmitic and stearic acid), SCD-1 (stearoyl-CoA desaturase, synthesizes palmitoleic and oleic acid), and GPAT-1 (glycerol-3-phosphate acyltransferase, preference for saturated FAs) enzyme activity [21]. This is an advantage of LINEX, which can depict relations of lipids also based on FA metabolism. The example also shows the importance of coverage of the lipidome. The more species available, the better connections between lipids can be inferred, ultimately helping to understand lipid metabolic alterations. The particular example lacks lyso-glycerophospholipids, which play a central role in the metabolism. Many lipids remain unconnected in this example or form components of less than four lipids, which makes the biological interpretation of the lipidome in the network context challenging (Figure A4).

In the previous example on the lipidome of colorectal cancer patients, one GPL component could be observed. Based on the data of Tu et al. [19], multiple such components can be found. The two biggest components can be seen in Figure 3. Both share a similar set of FAs from C16 to C22. The topological structures of both components also show similarities. Many triangles of PC, PE and PS species can be found, which share the same FA signature and are converted into each other by headgroup modifications (e.g., PC(18:0_18:1), PE(18:0_18:1), PS(18:0_18:1) or PC(18:0_20:4), PE(18:0_20:4), PS(18:0_20:4)). In some cases, additional connections to phosphatic acid (PA) or phosphatidylglycerol (PG) can be found. Other GPLs are connected purely via FA reactions (e.g., PE(22:5_22:6)). This pattern shows that certain FA combinations for GPLs seem favorable for enzymatic reactions, because they do not only occur in pairs but directly for up to five different lipid classes, which can be converted into each other.

Tu et al. [19] reported an overall decrease of GPLs and increase of sphingolipids and neutral lipids. With LINEX, we could visualize this trend on the whole lipidome (Figure A5). The global changes from the 4 week to the 12 week measurements were specific on the molecular species level, with small fold changes from 12 to 24 week old mice. The next change from 24 to 32 week probes showed the previously mentioned effect clearly with the GPL components being mainly decreased (red) and the rest mainly increased (blue). Interestingly, the ether lipids increased and therefore behaved opposite to the other GPLs. Finally, the comparison of 32 to 52 week old mice showed a similar pattern as the previous comparison, but with increased fold changes, especially in highly connected GPL such as PE(18:1_18:1), PC(16:0_20:4) or PE(22:4_22:6).

### 2.3. Healthy Human Reference Plasma Lipidome in Aging

As a third example, we are showcasing plasma lipidome data from a human reference population presented in Kyle et al. [22], which comprises 136 samples and 302 lipids, mostly identified as molecular species. All patients do not suffer from any diagnosed disease and represent the United States population in terms of age and sex distribution. To enable statistical comparisons, we grouped the patients by age (see Section 4 for details) and investigated the changes of the lipidome from young to old.

**Figure 4:**
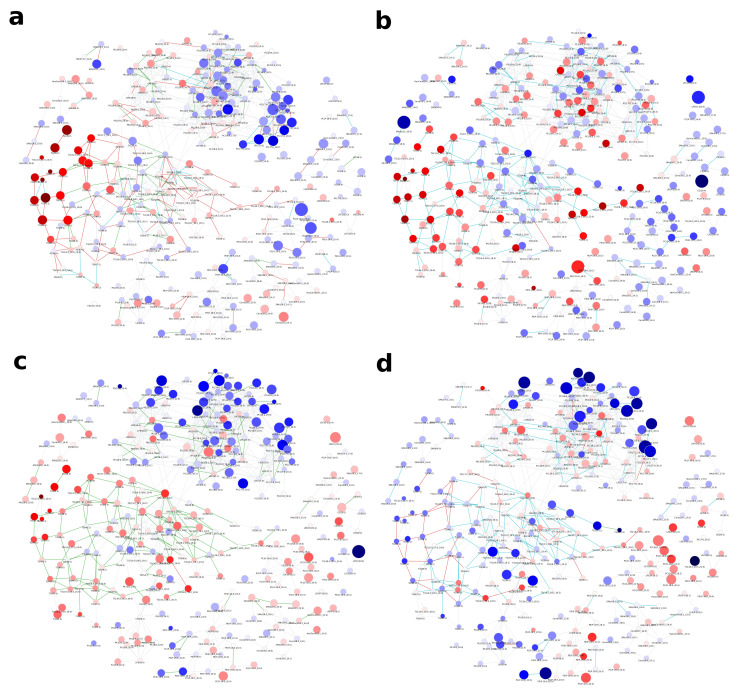
Global age-related plasma lipidome changes in a healthy human reference population from Kyle et al. [22]. Node colors represent log fold-changes with blue being negative, i.e., lower in the first condition, and red being positive. Node sizes are proportional to -log10(FDR) values. Edge colors indicate changes in correlation values between the respective conditions. For edge color groups see legend in Figure 2b. (**a**) Toddler vs. Child (**b**) Child vs. Teenager (**c**) Teenager vs. Adult (**d**) Adult vs. Elder.

Many edges in the network (Figure 4, interactive network: Supplementary Data 3) show non-statistically significant correlations in any of the age groups, as indicated by the large fraction of gray edges, especially in the area rich in PCs and PEs in the upper right part of the lipid network (compare Figure A6). Those areas, which show statistically significant correlations, do so in half of the groups, namely at the ‘Toddler’, ‘Child’ and ‘Elder’ stage. While these reactions affect PCs and PEs with a variety of molecular compositions, most of these reactions are FA related, which becomes especially clear for PC species with odd-chain FA on the lower right side of the subnetwork. Interestingly, many lipids in this subnetwork show differential abundances between toddlers and children (Figure A6a), which is accompanied by a higher density of strong correlations. For comparisons including young adults (Figure A6c,d), both the number of lipid species with a higher probability of being different between sample groups and the number of edges with changes in correlation are much lower in this area of odd-chain PCs. Considering the general structure of the subnetworks shown in Figure A6, these two groups show an interesting behavior with respect to the position of lipid species with high absolute fold changes, which are located mostly on the outside of the network, corresponding to lower node degree and betweenness centrality. Most changes in correlation, however, are happening in the inner part around higher connected nodes, especially lyso-species. A possible explanation for this phenomenon is that changes in the center of the network are propagated to more peripheral parts, while intermediate nodes stay nearly unaffected in their abundances, as reactions producing and transforming them are changing their activities to the same degree.

In contrast to the part of the network shown in Figure A6, the subnetwork depicted in Figure A7 mainly comprises TG species and is only lightly connected. This is possibly due to few reported DG species, which would be connected to multiple TGs similar to LPC species connecting PCs. Considering all four age comparisons ((i) Toddler vs. Child (ii) Child vs. Teenager (iii) Teenager vs. Adult (iv) Adult vs. Elder), most edges are either statistically significantly correlated in multiple comparisons or in none. This indicates constant metabolic activities shared across different age stages. Generally patients grouped as children, teenagers and young adults (see Section 4 for details) only show minor differences in TG levels (Figure A7b,c).

Investigating the changes from toddler to child in Figure A7a, shows that most TGs, which are differentially abundant, exhibit a chain length of 44 to 48 and 0 to 3 double bonds. On the one hand, most of these lipids are connected by edges representing strong correlations in both age groups. On the other hand, connections to unchanged lipids are mostly connected via edges that are only significant in the children group and represent FA elongations. As most of the species are only identified as sum species, potential FA-specific patterns cannot be observed. However, because the described changes apply to a very limited set of total chain lengths, FA-specific elongation patterns may play a major role in changing TG levels between toddlers and children.

For the comparison of adults to elder (Figure A7d), the previously described TG species are not differentially abundant, even though they are strongly correlated with each other. However, the few species with low *p*-values in the subnetwork comprise longer fatty acyls (a sum of 54 to 58 hydrocarbons), are more unsaturated (6 to 11 double bonds), and are located in two separate areas of the subnetwork. These lipids are sequentially connected via edges of the same type of correlation change (“ssignificant to insignificant”, referring to a statistically significant correlation in younger adults between two lipids, which is not statistically significant in older adults), e.g., TG(58:9), TG(58:10) and TG(58:11).

## 3. Discussion

Existing bioinformatics tools for lipidomics data analysis are mainly based on the lipid class metabolism, ontologies, the chemical space or correlations. With LINEX, a new type of analysis for lipidomics is available. We combined established statistical measures as already used in other lipidomics analysis approaches such as lipidr [1] and functional associations between lipids. The tool BioPAN [14] offers an analysis of lipid networks and aims to find active reaction chains. LINEX takes a different approach and focuses on visualizing statistics on networks and hence revealing global trends of the lipidome and local shifts of lipids through metabolic reactions. The LUX Score [15] also visualizes global alterations of the lipidome but does not show functional associations between lipids as LINEX does.

We applied LINEX to publicly available lipidomics data and were able to reveal new insights into the regulation of lipid metabolism in addition to the originally reported ones showing the advantages of a combined lipid network analysis for the biological interpretation of lipidomics experiments. Going beyond statistical comparisons of individual lipids, but considering functional associations between lipids together with correlations and a differential analysis of sample groups, we move towards a systems biological approach for the analysis of complex lipidomes.

With its versatile visualization options, LINEX offers lipid researchers the possibility to investigate lipidome changes on a global scale while also revealing specific local associations of lipids. Furthermore, the possibility to visualize changes in (partial) correlations between lipid pairs along with reaction types allows for a more holistic view on enzymatic changes affecting lipid metabolism to develop hypotheses about biological mechanisms. The visualized networks can be downloaded and shared as fully interactive standalone files.

As with all correlation analyses, LINEX can suffer from induced spurious correlation through indirect effects. Especially in the case of unmeasured reaction partners, both correlations and partial correlations are subject to possible false-positives. Therefore, results based on these metrics should always be interpreted with caution. Beyond the issue of spurious, undetectable lipids and low coverage can limit the interpretability of LINEX results, as important connections between different parts of the network may be missing. Future work on lipid metabolic networks has to aim at reducing the impact of these effects on data interpretation and the selection of putatively interesting subnetworks.

A particular challenge is the multi-specificity of many enzymes catalyzing lipid metabolic reactions, meaning they can catalyze conversions of multiple molecular lipid species belonging to the same lipid class. Hence, lipid metabolic networks have to be generated specifically for each dataset. This makes the workflow for lipid-metabolic networks fundamentally different to working with PPI or GR networks. Dedicated algorithms such as KeyPathwayMiner [3,4], DOMINO [5] or HotNet2 [6] perform an enrichment of deregulated genes on the whole network of possible interactions. However, with lipid species networks, the networks themselves carry information about the composition of the lipidome and its associations. Therefore, a direct application of common network enrichment tools for other biological networks is not possible. With the availability of molecular reaction networks by LINEX, we enable a combined analysis of lipidomics data and provide a basis to develop algorithms specifically for lipid networks, which integrate network (topological) approaches with statistical techniques. They hold the potential to associate changes in individual lipid species with global patterns in the lipid reaction network, thereby allowing them to go beyond pathway enrichment algorithms. This lays the foundation for further improvements in the analysis of lipid metabolic networks, integrating biochemical and statistical measures. With such approaches, the discovery of condition-specific network motifs will be possible. These motifs can then be used to define disease (sub-)types and to link conditions similar in their molecular lipid network patterns.

LINEX can be used to compare multiple conditions and switch between different network views to investigate systemic trends of lipidome changes. The versatility of LINEX allows users to create dataset-specific lipid-reaction networks, visualize and analyze the networks utilizing topological and statistical properties, as well as a standardized lipid class color scheme, and adapt the analysis to specific organisms, compartments or conditions, without requiring any programming knowledge, making it accessible not only to bioinformaticians but all lipidomics researchers. LINEX provides a novel view on the lipidome and can help to mechanistically understand remodeling of the lipidome. It can assist the community in mechanistic interpretation of lipid alterations and hypothesis generation.

## 4. Materials and Methods

### 4.1. Webtool

The LINEX web tool was implemented in python using the Django web framework. It is publicly available at https://exbio.wzw.tum.de/linex/ (accessed on 27 July 2021). The code is available at https://gitlab.lrz.de/lipitum-projects/linex (accessed on 27 July 2021). Interactive network visualizations were generated using the visjs-network library along with utilities from the pyvis [23] package. To achieve simple portability to other platforms with all dependencies, LINEX is running in a Docker environment and can be deployed locally.

### 4.2. Lipid Name Conversion

Lipidomics data often uses different lipid naming conventions. LINEX uses Lipid LynxX [20] to convert and standardize lipid names in order to recognize them. All lipids recognized by Lipid LynxX can be used by LINEX, if lipid class information and lipid class conversions are available. If they are not available by default, they can be extended by the user.

### 4.3. Dynamic Network Creation

**Figure A1:**
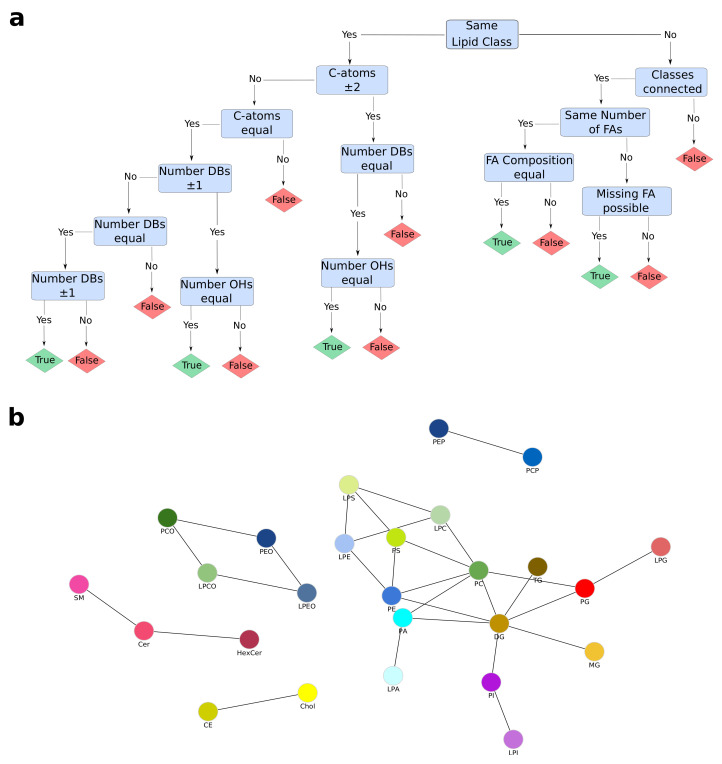
LINEX Default Reaction Rules. (**a**) Decision workflow for lipid connections with default fatty acid reaction rules. Due to the internal logic, lipid classes with different numbers of fatty acids have to have the same head group if they are connected. (**b**) Default lipid class connections. PEP: PE—Plasmalogen; PCP: PC—Plasmalogen.

**Figure A2:**
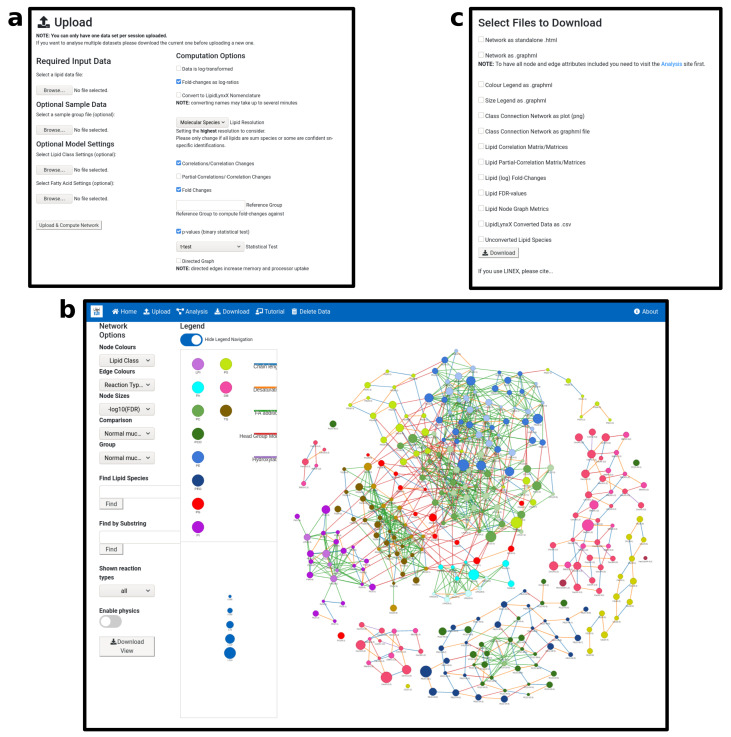
Main interfaces of the LINEX web-app. (**a**) Upload of lipidomics data with optional group labels for samples. Statistical methods can be selected for the visualization on the resulting network. Additionally, information about metabolic reactions and lipid classes can be uploaded to extend the network. (**b**) Analysis page. Here, the lipid networks can be interactively investigated and statistical or biochemical properties can be shown. (**c**) Download page. The network can be downloaded including all computed statistical measures.

**Figure A3:**
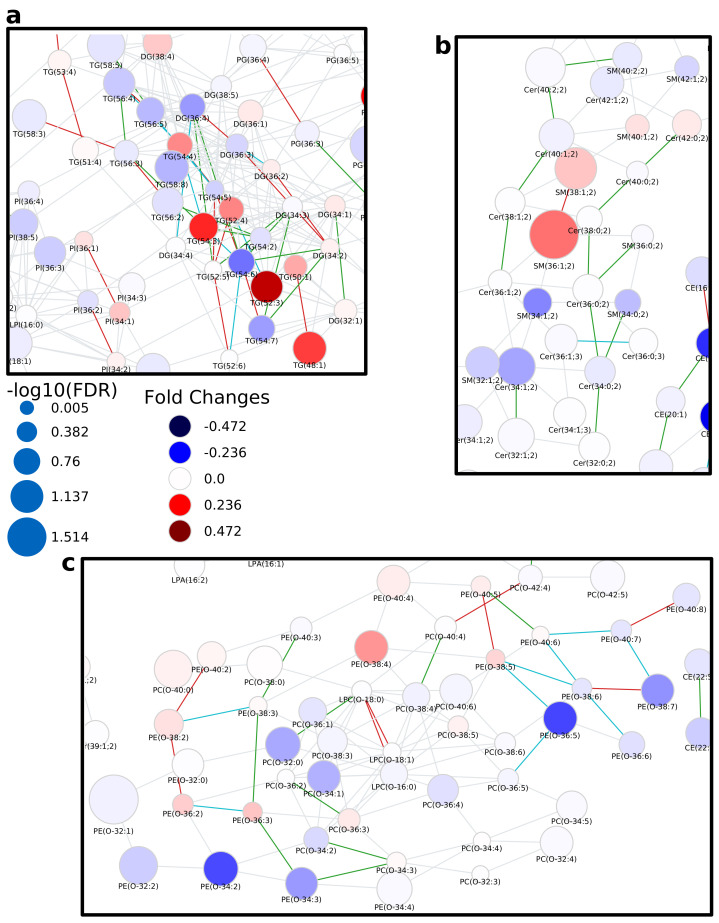
Detailed views on subnetworks of the lipidomics data of Wang et al. [16] showing the metabolism of (**a**) TG and DG, (**b**) ether lipids, and (**c**) sphingolipids. The full network can be seen in Figure 2. Nodes are colored by fold change and node size is scaled by−log10 of multiple testing corrected *p*-value. Edges are colored by correlation changes (see Figure 2).

**Figure A4:**
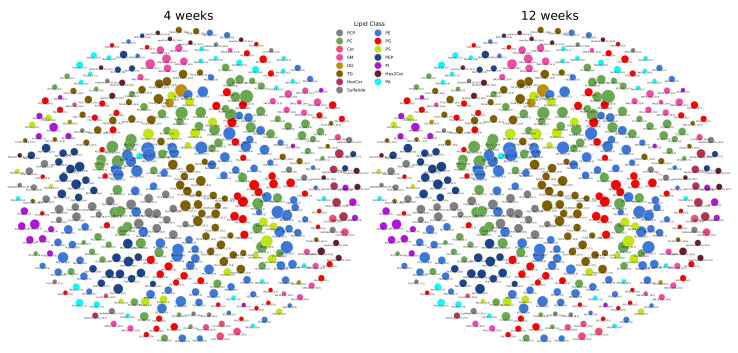
Lipid networks of the lipidomics data from Tu et al. [19]. Nodes are colored by lipid class and edges show correlations between lipids for each mouse age group. Significant and negative correlations are blue, significant and positive correlations red, and insignificant correlations gray. Other time points show similarly less significant correlations (not shown here).

**Figure A5:**
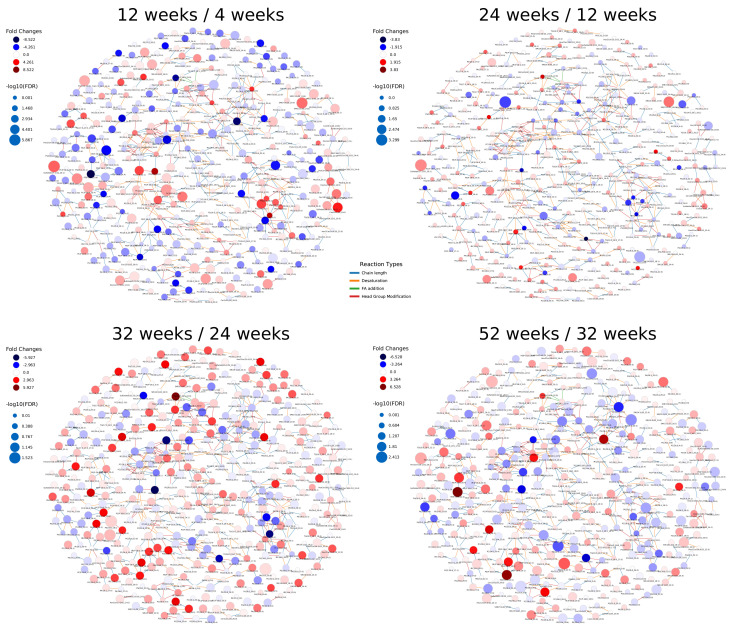
Fold changes of lipids visualized on lipid networks of the lipidomics data from Tu et al. [19]. Node size scaled by negative log10 of the *p*-values for comparison between healthy and cancer tissue. Lipids are colored by log fold change between mouse brain age groups. Blue indicates negative fold changes and red positive fold changes (e.g., higher levels in 12 weeks compared to 4 weeks are red). Edges are colored by reaction type. Chain length modification (blue), desaturation (orange), fatty acid addition (green) and head group modification (red).

**Figure A6:**
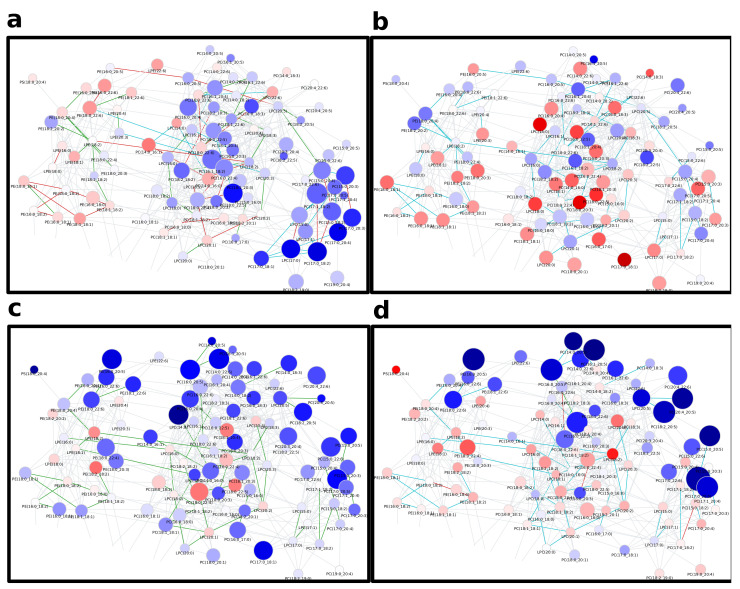
Detailed view on the PC/PE subnetwork from Kyle et al. [22] comparing (**a**) Toddler to Children, (**b**) Children to Teenager, (**c**) Teenager to Young Adults and (**d**) Young Adults to Older Adults.

**Figure A7:**
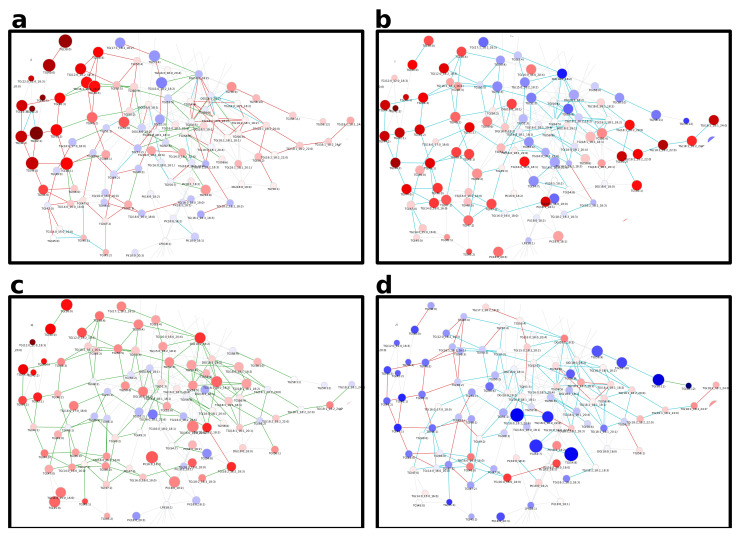
Neutral lipid subnetwork based on Kyle et al. [22] comparing (**a**) Toddler to Children, (**b**) Children to Teenager, (**c**) Teenager to Young Adults and (**d**) Young Adults to Older Adults.

The inference of lipid metabolic networks in LINEX is implemented in a modular way by splitting transforming reactions into two broad categories: class or headgroup-related transformations and fatty acid-related (FA-related) transformations. Two given lipid species are connected in the network if they either share all their FA(s) and their headgroups are connected by a reaction, or if both lipids have the same headgroup and exactly one FA pair is transformable, according to a set of input rules. If two lipids from different classes only differ in the number of FAs, e.g., a PC and a LPC, a connection is drawn if the “larger” (PC) lipid species contains all FAs present in the “smaller” (LPC) lipid and the missing FA is in a user-defined pool of possible FAs. The decision process with pre-defined FA rules is depicted in Figure A1a. Additionally, FA reactions are evaluated (elongation, desaturation and oxidation), connecting lipids of the same class if they differ in a chain length of two, a desaturation or oxidation (on the molecular species level this is considered for individual FAs). While this type of inferred connection is based on biochemical reactions, it only represents a heuristic. All edges of this type can interactively be hidden with one click. Further details for matching between lipids of different structural resolutions with examples can be found in Appendix B.

Due to the nature of the matching procedures, it is not possible to cover many-to-many reactions such as the modification of a ceramide with a phosphocholine group from a phosphatidylcholine to a sphingomyelin and a diacylglycerol.

Default rules for both lipid class reactions and FA reactions are available. The default lipid classes and their connections are shown in Figure A1b. Because of the versatility of the implementation, user-defined customization to any desired condition and organism are possible for both sets of rules. Furthermore, it is possible to manually customize enzyme annotation for all headgroup modifying reactions.

LINEX can handle three levels of FA resolution, sum composition, molecular species and sn-specific lipid annotations, but profits from identification of all FAs, due to higher specificity of the assigned edges. In order to utilize the maximum amount of information, mixed identification levels within a dataset are allowed. When matching species on sum composition level to species of higher structural resolution, the list of allowed FAs (Table A1) is used to determine whether a FA addition is possible under the given conditions. The only requirement for using LINEX is a lipid nomenclature compatible with Lipid LynxX [20], as internal lipid mapping depends on a unified nomenclature.

### 4.4. Lipid Class Color Scheme

We developed a color scheme to color lipids based on their class. This scheme is available in Supplementary Data 4 and on the linex website: https://exbio.wzw.tum.de/linex/download (accessed on 27 July 2021). It supports colors for 46 common lipid classes. Groups of lipids have similar colors, with lyso-species being brighter and ether classes darker. Colors are available as hex codes.

### 4.5. Statistical Methods

For analyzing changes between sample groups, multiple statistical measures are included, which can be separated into lipid species, i.e., nodes, specific and reaction, i.e., edge, specific metrics.

To compare lipid abundances, (log) fold-changes and binary statistical tests are available. End-users can choose between parametric (*t*-test) and non-parametric (Wilcoxon signed-rank test [24]) depending on their data distributions. All *p*-values are automatically reported as Benjamini-Hochberg corrected False Discovery Rates (FDR) [25]. These can be visualized as node color or size.

Additionally, three theoretical graph measures are computed for each note, namely degree, betweenness centrality [26] and closeness centrality [27]. These are, in contrast to the above metrics, independent of sample groups and visualized as node size or color.

Edge-related measures are based on correlations and partial-correlations. In order to compare two groups, (partial) correlation changes are sorted into five discrete groups, which represent whether the correlation between two lipids stayed (in-)significant, turned (in-)significant or changed its sign. In the network visualization, they are represented by the coloring of edges.

All statistical measures were computed using scipy [28] and scikit-learn [29]. For graph-related measures, the NetworkX [30] package was used.

LINEX does not provide data pre-processing options. Therefore, input data has to be readily processed (sample normalization, batch correction, normalization to internal standards or log-transformation). Future updates will be announced on the website: https://exbio.wzw.tum.de/linex/ (accessed on 27 July 2021).

### 4.6. Experimental Data Processing

For the evaluation, publicly available lipidomics datasets were used. The data from Wang et al. [16] was reformatted and lipid names converted with Lipid LynxX [20]. No further modifications were done to the quantified measurements. Lipidomics data from Tu et al. [19] was downloaded from the MetaboLights database [31] (Study ID: MTBLS562 and MTBLS495). Prior to uploading the data, reported as peak areas, it was quotient-normalized [32] and generalized log2 transformed. Healthy human reference population data of the plasma lipidome was taken from Kyle et al. [22]. Unsupported lipid classes, namely Sulfatide and Carnitine, two Endocannabinoids and Co-Enzyme Q10 were removed, and LPE-P was manually added to the lipid class settings file. Three ceramide species were measured in positive and negative mode. For these, only the negative mode information was used. Lipidomics data were downloaded from the MassIVE repository at https://doi.org/10.25345/C5P11F (MSV000085508; accessed on 27 July 2021). Patient metadata used can be found on figshare [33]. In order to compare age-related changes, patients were grouped into 4 groups. Toddler: 0 to 36 months; Child: 4–12 years; Teenager: 13–19 years; Adult: 20–49 years; Elderly: 50–81 (old patient).

## Data Availability

The developed software is open source. The source code is available at: https://gitlab.lrz.de/lipitum-projects/linex (accessed on 27 July 2021). For the analysis, publicly available lipidomics data was used (See methods section).

## Supplementary Materials

The following are available online at https://www.mdpi.com/article/10.3390/metabo11080488/s1, Supplementary Data 1: Interactive HTML of the network shown in Figure 2, Supplementary Data 2: Interactive HTML of the network shown in Figure 3, Supplementary Data 3: Interactive HTML of the network shown in Figure 4, Supplementary Data 4: Lipid Class Color Scheme.

## Abbreviations

FA	fatty Acid
GPL	glycerophospholipid
GR	Gene Regulatory
LGPL	lyso-glycerophospholipid
LPC	lyso-phosphatidylcholine
LPE	lyso-phosphatidylethanolamine
LPI	lyso-phosphatidylinositol
MS	Mass Spectrometry
PA	phosphatic acid
PC	phosphatidylcholine
PE	phosphatidylethanolamine
PEO	phosphatidylethanolamine Ether
PG	phosphatidylglycerol
PI	phosphatidylinositol
PPI	Protein-Protein Interaction
PS	phosphatidylserine

## Appendix A

**Table A1:** LINEX Default Fatty Acids. This list is used when lipids from different classes that only differ in the number of FAs are matched. Users can customize this list for their specific experimental conditions.

Saturated FAs	Monounsaturated FAs	Polyunsaturated FAs
14:0	16:1	18:2
15:0	18:1	20:2
16:0	20:1	20:3
17:0		20:4
15:0		20:5
20:0		22:4
		22:5
		22:6
		24:6

## Appendix B

In order to give a better intuition on how the rules work, we want to give three examples representing the basic types of reactions possible based on molecular species annotations.

PC(16:0_18:0)—PC(18:1_16:0): Both lipids share the same headgroup and have the same number of FAs. Therefore, the only possible reaction can be on FA level. Since 16:0 is shared in both, the remaining FAs need to be transformable. According to the default rules (Figure A1a), 18:0 → 18:1 fulfills the criteria for a desaturation, because the number of carbon atoms as well as the number of hydroxy groups stay the same, while the number of double bonds is changed by exactly one. As such fatty acid modifications are not known for esterified fatty acids, this edge represents a heuristic rather than a direct biochemical reaction. Users can remove all edges of this type in the interactive network visualization.

DG(16:0_18:0)—TG(18:1_18:0_16:0): While these lipids share the same headgroup they differ in the number of FAs. The first step in the further workflow is now to check whether the FAs in the DG, the species with fewer FAs, are both present in the putative reaction partner. As this is the case, we know that DG(16:0_18:0) and TG(18:1_18:0_16:0) are connected via the addition of an 18:1 FA. If these lipids were given as sum species, the difference between their sum compositions—34:0 and 52:1, respectively—would have been used to find the missing FA and a subsequent check of whether the resulting FA 18:1 is in the list of allowed FAs (see Table A1 for the default values) would have decided over whether the reaction is considered possible or not.

PE(16:0_18:0)—PC(16:0_18:0): The two species are composed of different headgroups; hence, the only possible reaction is a headgroup modification. For such a reaction, the lipids need to have the exact same FA composition. On sum species level, this requirement is loosened to both lipids having to have the same number of FAs and the same sum composition. Subsequently, the lipid class connection table (Figure A1b) is queried to validate whether a reaction transforming one headgroup into the other exists. Because this is the case, based on default settings, PE(16:0_18:0) and PC(16:0_18:0) are connected in the network.
