# Supplementary material for: Investigating Global Lipidome Alterations with the Lipid Network Explorer
Source: Metabolites. 2021 Jul 28;11(8):488. doi: 10.3390/metabo11080488 (PMC8398636; doi:10.3390/metabo11080488)
Supplement: Supplementary file 1 [file metabolites-11-00488-s001.zip › SupplementaryData1_LINEX_network_wang2020.html]

##### Network Options

**Node Colours**

Lipid Class
Desaturation
Chain Length
C Index
DB Index
-log10(FDR)
Fold Changes
Closeness Centrality
Betweenness Centrality
Degree
**Edge Colours**

Reaction Types
Correlations
Correlation Changes
**Node Sizes**

-log10(FDR)
Fold Changes
Chain Length
Closeness Centrality
Betweenness Centrality
Degree
DB Index
Desaturation
C Index
**Comparison**

Normal mucosa\_Tumor
**Group**

Normal mucosa
Tumor
**Find Lipid Species**


Find
**Find by Substring**

Find

  
  
**Shown reaction types**

all
only Class reactions
only FA reactions

  
  

**Enable physics**

##### Legend

Hide Legend Navigation

##### 

0%
