# Supplementary material for: Investigating Global Lipidome Alterations with the Lipid Network Explorer
Source: Metabolites. 2021 Jul 28;11(8):488. doi: 10.3390/metabo11080488 (PMC8398636; doi:10.3390/metabo11080488)
Supplement: Supplementary file 1 [file metabolites-11-00488-s001.zip › SupplementaryData2_LINEX_network_tu2017.html]

##### Network Options

**Node Colours**

Lipid Class
Desaturation
Chain Length
C Index
DB Index
-log10(FDR)
Fold Changes
Degree
Closeness Centrality
Betweenness Centrality
**Edge Colours**

Reaction Types
Correlations
Correlation Changes
**Node Sizes**

-log10(FDR)
Fold Changes
Degree
DB Index
Chain Length
Closeness Centrality
Desaturation
C Index
Betweenness Centrality
**Comparison**

0-52W\_1-32W
0-52W\_2-24W
0-52W\_3-12W
0-52W\_4-4W
1-32W\_2-24W
1-32W\_3-12W
1-32W\_4-4W
2-24W\_3-12W
2-24W\_4-4W
3-12W\_4-4W
**Group**

0-52W
1-32W
2-24W
3-12W
4-4W
**Find Lipid Species**


Find
**Find by Substring**

Find

  
  
**Shown reaction types**

all
only Class reactions
only FA reactions

  
  

**Enable physics**

##### Legend

Hide Legend Navigation

##### 

0%
