# Supplementary material for: Investigating Global Lipidome Alterations with the Lipid Network Explorer
Source: Metabolites. 2021 Jul 28;11(8):488. doi: 10.3390/metabo11080488 (PMC8398636; doi:10.3390/metabo11080488)
Supplement: Supplementary file 1 [file metabolites-11-00488-s001.zip › SupplementaryData3_LINEX_network_kyle2021.html]

##### Network Options

**Node Colours**

Lipid Class
Desaturation
Chain Length
C Index
DB Index
-log10(FDR)
Fold Changes
Betweenness Centrality
Degree
Closeness Centrality
**Edge Colours**

Reaction Types
Correlations
Correlation Changes
**Node Sizes**

-log10(FDR)
Fold Changes
C Index
Betweenness Centrality
DB Index
Desaturation
Degree
Closeness Centrality
Chain Length
**Comparison**

Child\_Old Adult
Child\_Teenager
Child\_Toddler
Child\_Young Adult
Old Adult\_Teenager
Old Adult\_Toddler
Old Adult\_Young Adult
Teenager\_Toddler
Teenager\_Young Adult
Toddler\_Young Adult
**Group**

Child
Old Adult
Teenager
Toddler
Young Adult
**Find Lipid Species**


Find
**Find by Substring**

Find

  
  
**Shown reaction types**

all
only Class reactions
only FA reactions

  
  

**Enable physics**

##### Legend

Hide Legend Navigation

##### 

0%
